# Evidence of protection against clinical and chronic hepatitis B infection 20 years after infant vaccination in a high endemicity region

**DOI:** 10.1111/j.1365-2893.2010.01312.x

**Published:** 2011-05

**Authors:** Y Poovorawan, V Chongsrisawat, A Theamboonlers, G Leroux-Roels, S Kuriyakose, M Leyssen, J-M Jacquet

**Affiliations:** 1 Department of Pediatrics, Faculty of Medicine, Center of Excellence in Clinical Virology, Chulalongkorn UniversityBangkok, Thailand; 2 Center for Vaccinology, Ghent University and HospitalDe Pintelaan, Ghent, Belgium; 3 GlaxoSmithKline BiologicalsRixensart, Belgium

**Keywords:** efficacy, hepatitis B, vaccine

## Abstract

**Summary:**

Vaccination against hepatitis B virus (HBV) immediately after birth prevents neonatal infection by vertical transmission from HBV carrier mothers. There is an ongoing debate whether infant vaccination is sufficient to protect against infection when exposed to HBV later in life. We studied 222 Thai infants born to HBsAg −/+ and HBeAg −/+ mothers who were vaccinated with recombinant hepatitis B vaccine at 0-1-2-12 months of age. A subset of 100 subjects received a booster dose at age 5 years. Blood samples collected yearly for 20 years were examined for anti-HBs antibodies and serological markers of hepatitis B infection (anti-HBc, HBsAg, and in selected cases HBeAg, anti-HBe, HBV DNA). During the 20-year follow-up, no subject acquired new chronic HBV infection or clinical hepatitis B disease. During the first decade, possible subclinical breakthrough HBV infection (anti-HBc seroconversion) was only observed in subjects born to HBsAg +/HBeAg + mothers (6/49 [12.2%]). During the second decade, breakthrough HBV infections were detected in all groups (18/140 [12.8%]). Increases in anti-HBs concentrations that were unrelated to additional HBV vaccination or infection were detected in approximately 10% of subjects in each decade. Primary infant vaccination with a recombinant hepatitis B vaccine confers long-term protection against clinical disease and new chronic hepatitis B infection despite confirmed hepatitis B exposure. (http://www.clinicaltrials.gov NCT00240500 and NCT00456625)

## Introduction

Hepatitis B remains a serious global public health problem despite the availability of effective vaccines for several decades. It is estimated that two billion individuals have been infected with hepatitis B virus (HBV) worldwide and 360 million individuals have chronic HBV infection and are at risk of death because of liver cirrhosis and hepatocellular carcinoma [1].

Chronic HBV is the most likely outcome when infection is acquired perinatally [1]. If a mother is positive for both HBV surface antigen (HBsAg) and HBV e antigen (HBeAg), the risk of chronic infection is as high as 70–90%. Thus, prevention of infection at an early age is critical in preventing chronic carriage and subsequent morbidity and mortality associated with chronically evolving infection. Infant immunization against HBV has been recommended by the World Health Organization since 1988 [2].

Countries in South-East Asia have historically been regions of high HBV endemicity [3–5]. However, infant vaccination has been highly successful in preventing HBV infection and sequelae, including deaths because of fulminant hepatitis and hepatocellular carcinoma, in countries where it has been implemented [6–9]. In Thailand, nationwide infant vaccination against HBV began in 1992. At that time, the incidence of HBV carriage was as high as 12% in some groups [5]. Twelve years after onset of nationwide immunization, HBsAg seroprevalence in vaccinated 6 month to 18-year-olds had dropped from 4.3% to 0.7% [7]. This reduction has been maintained, with anti-HBc seroprevalence also decreasing from 15.8% to 2.9% [7]. The risk of hepatocellular carcinoma is significantly lower in HBV-vaccinated children in Thailand, than in unvaccinated children [10].

The success of infant vaccination programmes in preventing vertical transmission of HBV during infancy and early childhood is well established [6]. Yet, less is known about long-term protection following infant vaccination in high endemicity countries. The development of clinical or chronic hepatitis B after completion of primary vaccination in infancy has not been described [11]. However, most follow-up studies of vaccinated cohorts rely on isolated ‘serological snapshots’ taken many years apart to identify exposure or infection. In a serological follow-up study of infants from Thailand, a high endemicity region of South-East Asia, who were vaccinated against HBV in infancy, we assessed serological parameters of HBV infection yearly for 20 years. This study provides a dynamic description of serological profiles in vaccinated subjects over time.

## Materials and methods

### Subjects and study design

**Fig. 1:**
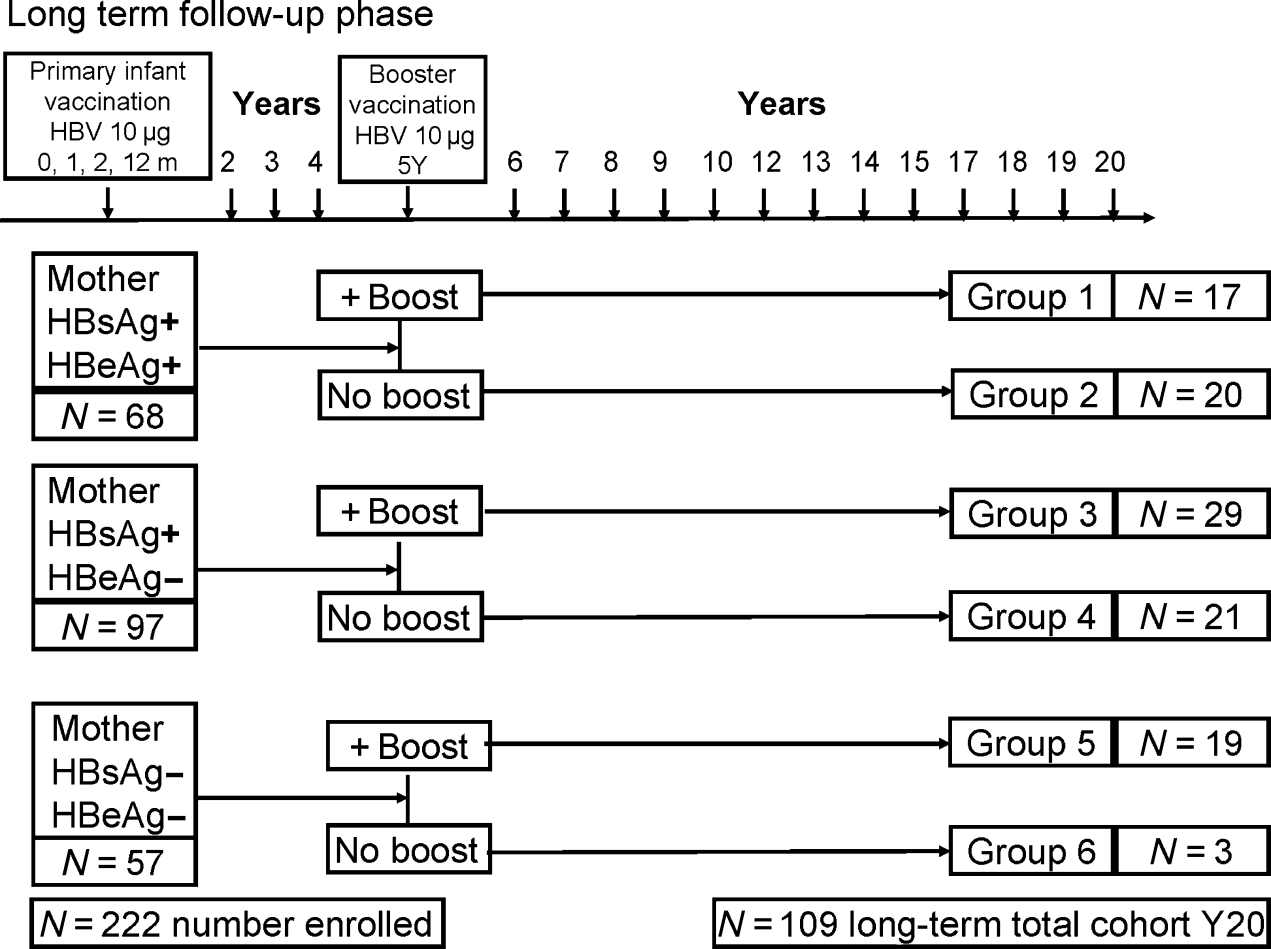
Study design.

The initial primary vaccination study was open labelled, with three groups based on the HBV infectious status of the mothers (HBsAg +/− and HBeAg +/−) and has been published previously [12,13]. Infants were vaccinated with recombinant hepatitis B vaccine (10 μg recombinant HBsAg; *Engerix*^tm^-B; GlaxoSmithKline Biologicals, Rixensart, Belgium) according to a 0-, 1-, 2- and 12-month schedule, beginning at birth. At 5 years of age, 100 subjects received a booster dose of the same vaccine [14]. Details of study groups and basis for their classification are presented in Fig. 1. The study was conducted between 1986 and 1988 at the Chulalongkorn University Hospital, Bangkok, Thailand.

All subjects were invited to attend yearly follow-up visits for 20 years (excluding Year 11 and Year 16 when no blood sampling was performed because of administrative delays in protocol development and approval) (study 100448, http://www.clinicaltrials.gov NCT00240500). At each visit, subjects were questioned about HBV clinical symptoms, and a blood sample was drawn. Informed consent was obtained from subjects before initiation of study procedures.

At Year 20, subjects who had anti-HBs below 100 mIU/mL at the previous follow-up were invited to participate in a challenge study in which they were given an additional dose of hepatitis B vaccine (*Engerix*^tm^-B, containing 10 μg HBsAg) to assess immune memory following exposure. Results of the long-term follow-up until Year 8 have been reported previously [14,15]. Humoral and cellular immune responses until Years 18–20 have been reported elsewhere [16]. Full immunological follow-up until Year 20 and response to the additional HBV dose have been reported in [17].

The studies were approved by the Institutional Ethic Committees, Faculty of Medicine, Chulalongkorn University and were conducted according to Good Clinical Practice, the Declaration of Helsinki (1996) and local rules and regulations of Thailand.

### Assessment of immunogenicity

All blood samples were tested for anti-HBs, HBsAg and anti-HBc. Between Year 12 and Year 15, a subset of selected samples in which HBsAg and/or anti-HBc was detected were tested for HBV DNA by polymerase chain reactions (PCR). During the final 4 years of follow-up, all serum samples positive for HBsAg and/or anti-HBc were further tested for HBV DNA, HBeAg and anti-HBe to further characterize the putative HBV infection. HBV DNA (core-pre-core-region) was measured using the COBAS Amplicor HBV Monitor (Roche Molecular Systems Inc., Branchburg, NJ, USA), [18], and HBV DNA (surface region) was detected by a PCR developed in house (amplification of 400 bp region using the primers: HBV-Fw 5′-TTATCGCTGGATGTGTCTGC-3′ and HBV-Rv 5′-CAGACTTGGCCCCCAATACC-3′). Liver enzymes were also measured during the final 4 years of follow-up, to evaluate clinical significance of breakthrough infection.

During the 20-year follow-up, some assays were replaced based on commercial availability and as new technologies became available. A list of the laboratory methods used over the duration of the study is given in Table 1.

**Table 1:** Laboratory assays

Marker Assay	Time point	Method	Test Kit/Manufacturer	Assay cut-off
Anti-HBs	Y0–Y13	RIA	AUSAB/Abott	1 mIU/mL
	Y14–Y18	EIA	AUSAB/Abbott	3.3 mIU/mL
	Y19–Y20	EIA	In house	3.3 mIU/mL
Anti-HBc	Y0–Y13	RIA	Corab/Abbott	+/−
	Y14–Y20	EIA	AxSYM CORE/Abbott	+/−
HBsAg	Y0–Y12	RIA	AusRIA/Abbott	+/−
	Y13–Y20	EIA	AxSYM HBsAg/Abbott.	+/−
Anti-HBe	Y16–Y20	EIA	AxSYM/Abbott	+/−
HBeAg	Y16–Y20	EIA	AxSYM/Abbott	+/−
HBV DNA (HBsAg)	Y12–Y15 subset	PCR	In house	+/−
	Y16–Y20	PCR	In house	+/−
HBV-DNA (HBsAg)	Y16–Y20	Sequencing	NA	NA
HBV DNA (Pre-core/core)	Y17	PCR	Cobas monitor/Roche	200 copies/mL
	Y18–Y20	PCR	Cobas monitor/Roche	316 copies/mL
AST	Y16–Y20	Kinetic UV assay	Roche Diagnostics	0–38 U/L
ALT	Y16–Y20	Kinetic UV assay	Roche Diagnostics	0–38 U/L

Y, year of follow-up; EIA, Enzyme-linked immunoassay; NA, not applicable; mIU/mL, milli International Units per millilitre; U/L, units per litre.

### Statistical methods

The clinical significance of HBsAg-positive and/or anti-HBc-positive samples was reviewed for each subject, taking into account: the presence of other HBV markers (anti-HBs, HBeAg, anti-HBe); the presence of HBV DNA (pre-core/core and surface antigen); serum transaminase activities (ALT/AST) together with the medical history of the subject and the presence of HBV markers at previous or subsequent time points. Based on these results, subjects were classified according to the following criteria:

- Chronic HBV infection: HBsAg+ AND anti-HBc+ at more than two consecutive time points;
- Clinical HBV infection: Clinical, serologically confirmed HBV infection;
- False positive: Single marker of HBV infection (HBsAg, PCR, HBeAg, anti-HBc) positive in one sample, AND all other markers negative in this sample, AND this and other HBV markers negative at consecutive time points;
- Possible subclinical breakthrough HBV infection: One or more HBV marker positive in one or more consecutive samples, excluding conditions for chronic HBV infection and false positive result, without any reported clinical symptoms of hepatitis;
- Isolated natural booster: ≥Fourfold increase in anti-HBs concentrations if <100 mIU/mL at previous time point, OR ≥twofold increase in anti-HBs concentrations if ≥100 mIU/mL at previous time point, AND all other serological markers for HBV infection negative at this time point.

This classification was performed separately for the first and the second decades. A subject was considered evaluable if results were available for at least two blood samples in a given decade. Subjects that acquired chronically evolving HBV infection in the perinatal period (*n* = 2) were excluded from further analysis.

## Results

### Study population

Two hundred and twenty-two infants were enrolled at the study onset, which was initiated in 1986. Of these, 109 subjects participated in the final follow-up visit 20 years later. At Year 20, the mean age of the subjects was 19.6 ± 0.49 years, and 49.5% of subjects were men. All subjects were of Asian or South-East Asian heritage.

### Incidence of HBV infectious events over time

Perinatal HBV infection that evolved towards chronicity was acquired at birth by two subjects who did not respond to the primary vaccination course. These subjects were described previously [13]. During the 20-year follow-up, none of the remaining subjects acquired a HBV infection that evolved chronically (determined by the observation of HBsAg and anti-HBc seropositivity for more than two consecutive time points) (Table 2). None of the subjects reported clinical symptoms of HBV disease during the 20-year follow-up. None showed elevated ALT or AST during the study.

**Table 2:** Incidence of hepatitis B infectious events

	Mother HBsAg+ HBeAg+			Mother HBsAg+ HBeAg−			Mother HBsAg− HBeAg−		
			
Assessment per subject		Decade 1	Decade 2		Decade 1	Decade 2		Decade 1	Decade 2
									
Boosted at age 5	Group	*N* = 21 *n* (%)	*N* = 18 *n* (%)	Group	*N* = 45 *n* (%)	*N* = 48 *n* (%)	Group	*N* = 33 *n* (%)	*N* = 22 *n* (%)
False positive	1	0 (0.0)	2 (11.1)	3	0 (0.0)	4 (9.8)	5	1 (3.0)	2 (9.1)
Isolated natural booster response		3 (14.3)	5 (27.8)		2 (4.4)	4 (9.8)		1 (3.0)	3 (13.6)
Subclinical breakthrough HBV infection		2 (9.5)	4 (22.2)		0 (0.0)	4 (9.8)		0 (0.0)	1 (4.5)
Clinical HBV infection		0 (0.0)	0 (0.0)		0 (0.0)	0 (0.0)		0 (0.0)	0 (0.0)
New chronic HBV infection		0 (0.0)	0 (0.0)		0 (0.0)	0 (0.0)		0 (0.0)	0 (0.0)

Unboosted	Group	*N* = 28 *n* (%)	*N* = 21 *n* (%)	Group	*N* = 43 *n* (%)	*N* = 32 *n* (%)	Group	*N* = 10 *n* (%)	*N* = 6 *n* (%)
False positive	2	2 (7.1)	2 (9.5)	4	0 (0.0)	9 (28.1)	6	0 (0.0)	1 (16.7)
Isolated natural booster response		5 (17.9)	1 (4.8)		6 (14.0)	1 (3.1)		1 (10.0)	1 (16.7)
Subclinical breakthrough HBV infection		4 (14.3)	6 (28.6)		0 (0.0)	2 (6.3)		0 (0.0)	1 (16.7)
Clinical HBV infection		0 (0.0)	0 (0.0)		0 (0.0)	0 (0.0)		0 (0.0)	0 (0.0)
New chronic HBV infection		0 (0.0)	0 (0.0)		0 (0.0)	0 (0.0)		0 (0.0)	0 (0.0)

*N*, Number of evaluable subjects/group/decade; Note Group 6, 2nd decade *N* = 6, thus results should be interpreted cautiously.

During the first decade, possible subclinical breakthrough HBV infections, accompanied by the emergence of anti-HBc antibodies, were only observed in subjects born to mothers positive for both HBsAg and HBeAg (2/21 [9.5%] in group 1 and 4/28 [14.3%] in group 2).

During the second decade, possible subclinical breakthrough HBV infections were detected in all groups: 4/18 (22.2%) in group 1, 6/21 (28.6%) in group 2, 4/41 (9.8%) in group 3, 2/32 (6.3%) in group 4, 1/22 (4.5%) in group 5 and 1/6 (16.7%) in group 6.

Increases in anti-HBs concentrations, unrelated to additional HBV vaccination or HBV infection, were detected in 10% of subjects in the first decade and 10.7% in the second decade. If not related to vaccine administration, these are likely to have been caused by natural boosting.

False positive results (according to the definition mentioned earlier) were detected in 23 subjects. In most cases, this was an isolated HBsAg+ result that was most frequently observed with the AxSYM HBsAg (Abbott) assay used in the final years of the follow-up (Y13–Y20). All of the HBsAg results in these subjects were low values ranging between 2.02 and 3.63 (assay cut-off for positive >2 S/N ratio), suggesting these were indeed false positive results.

### Longitudinal assessment of possible breakthrough cases

**Fig. 2:**
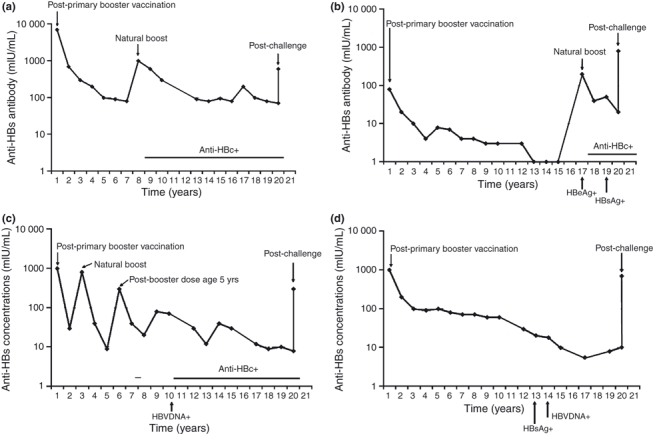
(a) Subject who seroconverted for antibodies against HBc during the follow-up period, with anti-HBs booster response. (b) Subject (not boosted at Year 5) with >1 serological marker indicative of hepatitis B infection, unaccompanied by anti-HBs booster response or emergence of antibodies to HBc. (c) Subject who developed detectable antibodies against HBc during the follow-up period without an anti-HBs booster response. (d) Response to possible subclinical breakthrough HBV infection in a low responder after primary and booster vaccination (no booster given at Year 5). In all graphs, the response to administration of a challenge dose of HBV vaccine at Year 20 is evident.

Twenty-four subjects with possible subclinical HBV breakthrough infections were identified. Individual analysis of serological results allowed further differentiation of these subjects into three subgroups showing different serological profiles:

Eleven subjects seroconverted for anti-HBc antibodies during the follow-up period and showed an anti-HBs booster response compatible with a self-limited breakthrough infection. An example of such a serological profile is provided in Fig. 2a. Interestingly, one of these subjects was a low responder to primary vaccination and the 12-month booster dose (<100 mIU/mL). This subject had undetectable anti-HBs antibodies from Year 13 onwards. However, upon exposure to the virus at 17 years of age, as indicated by emergence of anti-HBc antibodies, anti-HBs antibodies rose sharply (Fig. 2b).

Three subjects developed detectable antibodies against HBc during the follow-up period but showed no indication of an anti-HBs booster response (Fig. 2c).These three subjects responded well to the primary vaccination (post-primary anti-HBs concentration of 1096–6575 mIU/mL), and the detection of anti-HBc antibodies at multiple time points is a clear indication for exposure to the virus.

Finally, 10 subjects had more than one positive serological marker for HBV infection, or the same marker was detected at more than one time point. In these 10 subjects, neither an anti-HBs booster response, nor emergence of anti-HBc antibodies was observed after the apparent infection. This type of serological profile is illustrated in Fig. 2d. Isolated positive HBsAg samples were found in all these subjects, five had HBV DNA detected with values between 2730 and 10 730 copies/mL. One subject was also positive for HBeAg on one occasion.

## Discussion

Long-term serological follow-up studies after hepatitis B vaccination frequently assess the serostatus of individuals at infrequent, widely spaced intervals. These serological ‘snapshots’ may be subject to confounding factors including detection of false positives. This longitudinal study spanning 20 years provides a unique, dynamic view of the evolving serological status of subjects over time. This study shows the efficiency of infant HBV vaccination in preventing HBV infection, as well as the serological consequences of HBV exposure/infection for two decades after vaccination.

In total, 24/109 subjects (22%) showed strong serological evidence of exposure to HBV over the 20-year follow-up period. During the first decade, serological indications of transient asymptomatic HBV infections were only observed in subjects born to high risk families (mother positive for both HBsAg and HBeAg, groups 1 and 2). This is consistent with the notion that at a very young age, HBV infection is mostly transmitted through household contact. In the second decade, breakthrough infections were detected in 12.9% of subjects and occurred in all groups, possibly reflecting increased exposure outside the home linked to adolescent risk behaviours. In addition, isolated increases in anti-HBs antibody concentrations, indicative of natural boosting caused by exposure to HBV, were observed in approximately 10% of subjects in all groups each decade. This level of exposure is compatible with epidemiological data for HBV in Thailand; in a seroprevalence study, anti-HBc seroprevalence in (unvaccinated) 18- to 20-year-old Thai subjects was 21.7% [7]. Despite this high risk of exposure to HBV, none of the subjects acquired chronic HBV infection nor did any subject report clinical symptoms of HBV disease. These results therefore suggest that infant HBV vaccination protects against clinical hepatitis B disease and chronic carriage until at least 20 years of age.

Of the 24 individuals with evidence of possible subclinical breakthrough HBV infection (occult infection), 11 showed a typical response with emergence of anti-HBc antibodies and an anti-HBs booster response. Notably one subject had a low response to the primary vaccination course but still retained immunological memory to the virus 17 years later.

Atypical profiles were observed in 13 subjects. All three anti-HBc seroconverters without an anti-HBs booster response showed good anti-HBs responses to the primary vaccination course, as well as a response to a challenge dose administered 20 years later (Fig. 2c, other data not shown). The latter suggests that immune memory to the vaccine antigen was still present in these subjects and prevented HBsAg expression during the HBV infection to reach levels capable of boosting anti-HBs responses. In two of these subjects, HBV was also detected with PCR and/or HBsAg ELISA, making it unlikely that the anti-HBc conversion was aspecific. For neither of these subjects was the viral DNA successfully sequenced, making it impossible to ascertain whether these infections were attributed to mutated forms of the virus, of which the HBsAg was not recognized by the immune system. Importantly none of these subjects reported clinical signs of disease nor was an increase in liver enzymes detected.

Finally, the clinical interpretation of results from 10 subjects who were positive for HBsAg on one or more occasion or had other serological signs of HBV infection without developing detectable antibodies against HBc is less clear. An atypical presentation of breakthrough infection cannot be excluded; however, sample contamination or false positive results may also be responsible for these findings.

Previous follow-up studies in countries of high HBV endemicity have shown good protection against HBV carriage following infant vaccination with both plasma-derived and recombinant vaccines [19–22]. However, the importance of natural boosters in maintaining immunity, and the response of subjects on exposure to HBV, is not well described. In this study, the serological consequences of HBV exposure have been documented, and the ability of vaccinated subjects to clear HBV after subclinical infection has been demonstrated. These results are in line with those of a longitudinal study conducted in China in which yearly blood samples were collected from individuals vaccinated in infancy with either plasma derived or recombinant vaccines [23]. During the 22 years of the study, 23% had an anti-HBs booster response during the 22 years of the study, and no hepatitis B cases with evolving chronicity were documented [23]. Serological results that were not easily explained were also observed, with a small percentage of subjects becoming anti-HBc positive during the study without increase in anti-HBs.

In conclusion, primary infant vaccination with a recombinant HBV vaccine confers long-term protection against clinical disease and HBV infections that evolve towards chronicity, despite confirmed HBV exposure. Breakthrough infections became more frequent in the second decade of life, suggesting an increase in exposure and self-limiting infection with hepatitis B.

## Abbreviation

HBV: hepatitis B virus
